# Health literacy as an indirect pathway in the association between health promotion behaviors and social network addiction symptoms among adolescents

**DOI:** 10.3389/fpubh.2026.1897518

**Published:** 2026-10-07

**Authors:** Shaimaa Mohamed Amin, Sameer A. Alkubati, Halla Ali Abd El Hie Ali, Mahmoud Abdelwahab Khedr, Sally Mohammed Farghaly Abdelaliem, Nesreen AbdelMonaem AbouZeid, Ayman Mohamed El-Ashry

**Affiliations:** 1 Department of Community, Psychiatric, and Mental Health Nursing, College of Nursing, Qassim University, Buraydah, Saudi Arabia; 2 Department of Community Health Nursing, Faculty of Nursing, Damanhour University, Damanhour, Egypt; 3 Department of Nursing Services, Qassim University Medical City, Buraydah, Saudi Arabia; 4 Department of Medical Surgical Nursing, College of Nursing, University of Hail, Hail, Saudi Arabia; 5 Faculty of Nursing, Beni-Suef University, Beni-Suef, Egypt; 6 Department of Psychiatric and Mental Health Nursing, Faculty of Nursing, Alexandria University, Alexandria, Egypt; 7 Department of Nursing Administration, Faculty of Nursing, Alexandria University, Alexandria, Egypt; 8 Department of Nursing, Faculty of Allied Health Sciences, Kuwait University, Kuwait City, Kuwait; 9 Department of Medical and Surgical Nursing, College of Nursing, Princess Nourah bint Abdulrahman University, Riyadh, Saudi Arabia

**Keywords:** adolescent, Egypt, health literacy, health promotion, mediating effect model, social media use, social network addiction symptoms

## Abstract

**Background:**

Although health-promoting behaviors and health literacy may be protective factors against Social network addiction symptoms, little is known about the mechanisms underlying these interactions. This study aimed to investigate the relationship between health promotion and social network addiction symptoms among adolescents and to examine the mediating role of health literacy in this relationship.

**Methods:**

A cross-sectional descriptive study was conducted among 475 adolescents aged 15–18 years recruited from four secondary schools in Beni-Suef Governorate, Egypt, using a multistage random sampling technique. A demographic questionnaire, the Short-Form Adolescent Health Promotion Scale (SF-AHPS), the Health Literacy for School-Aged Children (HLSAC) scale, and the Social Network Addiction Scale (SNAS) were used to gather data between February and April of 2026.

**Results:**

The average scores were 30.04 ± 8.53 for social network addiction symptoms, 31.02 ± 4.85 for health literacy, and 70.61 ± 10.69 for health promotion. Health promotion was positively correlated with health literacy (*r* = 0.327, *p* < 0.0001) and negatively correlated with social network addiction symptoms (*r* = −0.117, *p* = 0.011). Health literacy was also negatively correlated with social network addiction symptoms (*r* = −0.229, *p* < 0.001). A cross-sectional statistical mediation model showed that health promotion was positively associated with health literacy (*β* = 0.299, *p* < 0.001), whereas health literacy significantly associated with lower social network addiction symptoms (*β* = −0.277, *p* < 0 0.001). While the indirect effect through health literacy was significant [*β* = −0.083, BootSE = 0.019, 95% CI (−0.122, −0.048)], suggesting a significant indirect association through health literacy, while the direct association was not statistically significant (*β* = −0.020, *p* = 0.664).

**Conclusion:**

Health literacy showed a significant indirect statistical association between health promotion and social network addiction symptoms among adolescents. Adolescents with higher health-promoting behaviors demonstrated better health literacy, which in turn was associated with lower levels of social network addiction symptoms.

## Introduction

Adolescence is a pivotal developmental period marked by rapid physical, cognitive, emotional, and social transitions. These developmental changes play a crucial role in shaping health behaviors, lifestyle choices, and overall wellbeing, with long-term implications for health outcomes throughout adulthood. As adolescents strive for greater independence and develop their personal identities, they establish behavioral patterns that may persist across the life course (1). Health-related habits formed during adolescence, including nutrition, physical activity, stress management, and social behaviors, play a substantial role in determining future health outcomes and quality of life (2). Consequently, promoting healthy lifestyles during adolescence has become a global public health priority (3).

In the last few years, digital technologies and social media platforms have become a big part of adolescents’ everyday lives (4). Social media platforms, including Instagram, TikTok, Snapchat, Facebook, and X, provide users with opportunities for communication, information exchange, social interaction, entertainment, and the development and maintenance of interpersonal relationships (4, 5). Adolescents are some of the most frequent users of social media around the world because they have a strong need to connect with others and express themselves (4). While social media can provide educational and social benefits, using it too much or without control can cause issues often referred to as social network addiction symptoms (6).

Social network addiction is characterized by excessive, compulsive, and maladaptive use of social networking platforms that can harm adolescents’ mental health, schoolwork, relationships, and daily functioning (7). Adolescents are especially at risk for this type of behavioral addiction due to their emotional sensitivity, the influence of friends, and the ongoing changes in their brain development that affect impulse control and how they respond to rewards (8). Excessive social networking use has been associated with sleep disturbances, anxiety, depression, reduced physical activity, poor academic achievement, and impaired social functioning among adolescents (3).

The growing prevalence of social network addiction symptoms among adolescents represents a significant public health concern worldwide (9). Recent evidence suggests that Social network addiction symptoms has increased substantially following the expansion of smartphone accessibility and internet connectivity, especially after the COVID-19 pandemic (9, 10). Adolescents in developing countries might encounter extra dangers because they have less access to digital health education, irregular supervision from their parents, and differences in health knowledge (11). Therefore, identifying protective factors that may reduce the likelihood of social network addiction symptoms is essential for enhancing the wellbeing of adolescent.

Health promotion is considered one of the most important determinants of adolescent wellbeing and healthy lifestyle development. Health-promoting behaviors encompass a range of actions, attitudes, and lifestyle practices aimed at maintaining and enhancing physical, psychological, and social wellbeing. These behaviors include healthy nutritional practices, regular physical activity, effective stress management, personal health responsibility, life appreciation, and the utilization of social support networks (12). Adolescents who take part in healthy activities are more likely to show better ways to handle stress, greater ability to bounce back from challenges, and stronger self-control skills. These positive traits can help keep them from developing unhealthy habits, like using social media too much (13).

Earlier research has indicated that adolescents who lead healthier lifestyles usually show fewer signs of behavioral addictions, such as internet addiction and issues with smartphone use (14, 15). Physical activity, effective stress management, and strong social support systems have been linked to better psychological adjustment and reduced dependence on online interactions. On the other hand, adolescents who do not take care of their health might turn to social media to help them deal with stress, feelings of loneliness, boredom, or emotional problems (13).

Health literacy has emerged as another critical factor influencing adolescent health outcomes and health-related decision-making (16). Health literacy refers to how well people can find, understand, judge, and use health information to make good choices about staying healthy and preventing illnesses (17). Among adolescents, health literacy is more than just being able to read. It also means being able to carefully think about health information found online, understand what they see on the internet, and take part in smart health practices as we spend more time in digital spaces (16).

Adequate health literacy among adolescents has been associated with healthier lifestyle practices, improved self-care behaviors, greater psychological wellbeing, and better use of health services (18). Adolescents with higher health literacy levels are more likely to take steps to prevent health issues, live healthier lives, and carefully think about possibly dangerous things they might find online (16). In contrast, having low health literacy can make it harder for adolescents to see the bad effects of using social media too much and can lessen their ability to control their behavior online (19).

Emerging evidence suggests that understanding health information might help protect people from harmful use of the internet and social media. Adolescents who have better health literacy skills might find it easier to manage their screen time, notice unhealthy behaviors online, and balance their time spent online with activities that promote good health offline (20). Furthermore, health literacy may support people manage their feelings and think clearly. These skills are important for lowering behaviors that lead to excessive or addictive use of social media (17).

Despite growing interest in how adolescents use digital technology, the connection between promoting health, understanding health information, and being addicted to social networks has not been studied enough. Most studies so far have looked at these factors on their own, and there has not been much research into how healthy behaviors might affect addiction to social networks (15, 16). Understanding the mediating role of health literacy may provide valuable insight into how healthy lifestyle practices contribute to reducing Social network addiction symptoms among adolescents.

The present study is theoretically grounded in Pender’s Health Promotion Model (HPM) and Bandura’s Social Cognitive Theory (SCT). These well-established theoretical frameworks provide a comprehensive foundation for understanding the multifaceted determinants of adolescent health behaviors and the dynamic interactions among individual, behavioral, and environmental factors that influence health outcomes. Pender’s Health Promotion Model highlights that a person’s experiences, how they think about things, their confidence in themselves, the influence of others around them, and the circumstances they find themselves in all work together to influence how they participate in activities that promote good health (21). This model suggests that adolescents who practice good health habits like managing stress, taking care of their health, staying active, and having social support are more likely to build better self-control skills and effective ways to handle challenges. This can help them avoid negative behaviors, such as spending too much time on social media (22). In parallel, Social Cognitive Theory proposes that behavior is influenced through reciprocal interactions among personal, behavioral, and environmental factors (23). Central to this theory is the construct of self-efficacy, which significantly influences an individual’s capacity for behavioral self-regulation, informed decision-making, and the effective management of health-related actions (24). Within the context of the current study, health literacy can be seen as a key mental tool that helps adolescents better assess information they find online, identify unhealthy behaviors related to digital use, and manage their time spent on social media. Health literacy is understood as a way that helps connect healthy habits to a decrease in social network addiction symptoms in adolescents.

In Egypt and other Middle Eastern countries, adolescents are experiencing rapidly increasing exposure to digital technologies and social networking platforms (25). However, evidence regarding the psychosocial and health-related determinants of social network addiction symptoms among Egyptian adolescents remains limited. Additionally, there have been only a few studies in the Arab region that looked into how health literacy affects the behavior of adolescents (25, 26). Exploring these relationships within the Egyptian cultural context is important for developing culturally appropriate health education and digital wellness interventions.

Therefore, the present study aimed to investigate the relationship between health promotion and social network addiction symptoms among adolescents and to examine the mediating role of health literacy in this relationship. Understanding these associations may contribute to the development of school-based health promotion programs and health literacy interventions designed to foster healthier digital behaviors and reduce the risk of social network addiction symptoms among adolescents.

## Methods

### Study design and setting

This study used a cross-sectional descriptive research design, which ensured that the STROBE checklist for cross-sectional studies was followed at every stage of the investigation. Four secondary schools in Egypt’s Beni-Sueif Governorate participated in the study. Beni-Suef Governorate is a heterogeneous sociodemographic setting that encompasses both urban and rural areas. The chosen schools are run by the Ministry of Education and cater to adolescents from a range of socioeconomic backgrounds.

### Sample size and study population

Adolescents aged 15–18 years, attending one of the chosen secondary schools in Beni-Suef Governorate during the study period, and consented to participate were included. Students with diagnosed cognitive, psychiatric or learning disorders that would affect the ability to understand and complete study instruments were excluded, as well as students who were not present during data collection or refused participation or parental consent was not acquired.

### Sample size

Sample size was determined using a dual approach. First, a minimum of 200 cases is recommended for path analysis using maximum likelihood estimation (27). Second, the required sample size for this study was determined *a priori* using G*Power version 3.1.9.4 (28). To align the calculation with the primary multivariate path analytic framework (mediation analysis via ordinary least squares regression), an *F*-test family for Linear Multiple Regression (Fixed model, *R*^2^ deviation from zero) was employed. Based on an assumed small effect size (*f*^2^ = 0.05), a significance level (*α*) of 0.05, and a high statistical power (1 − *β* of 0.95 across 5 structural associated factors), comprising the independent variable, the mediator, and three sociodemographic covariates (gender, mother’s education level, and home internet access), the analysis indicated that a minimum total sample size of 402 participants was required (with a critical *F* = 2.237, numerator df = 5, and denominator df = 396). Accordingly, a total of 475 participants were included in the present study, exceeding the minimum required sample size.

### Recruitment and sampling

Students from Beni-Suef Governorate were selected using a multistage sampling technique. The governorate includes eight educational districts, from which four districts were randomly chosen. One public secondary school was then selected from each district. Lists of students enrolled during the 2025–2026 academic year were obtained from the Student Affairs Department, and participants were randomly recruited from each school using a randomization program.

### Measurements

#### The demographic form

pretested sociodemographic data proforma was used to collect the details including age, sex, residence, and family profile of the participants. Family related information were father and mother working status, family type, parents’ educational level. The second form also included questions related to their digital access, specifically the predominant device used to access social media and whether there was household internet access.

#### Persian version of the short-form adolescent health promotion scale

The Short-Form Adolescent Health Promotion Scale (SF-AHPS), developed by Sarkhani et al. (29), was used to assess health-promoting behaviours among adolescents across six domains. The scale includes 21 items divided into six dimensions: nutrition (3 items), exercise (3 items), health responsibility (4 items), stress management (3 items), social support (4 items), and life appreciation (4 items). Items are rated on a 5-point Likert scale ranging from “never” 1 to “always” 5, with higher scores reflecting more frequent engagement in health-promoting behaviours. Total scores are calculated by summing the mean scores of the subscales, resulting in a possible range of 21 to 105. The scale has demonstrated excellent reliability, with a Cronbach’s alpha of 0.905 and McDonald’s omega of 0.904. In the current study, the internal consistency was confirmed with a Cronbach’s alpha of 0.813. Higher scores indicate better health promotion, whereas lower scores reflect a less favourable health-promoting status.

#### Health literacy for school-aged children (HLSAC) scale

The Health Literacy for School-Aged Children (HLSAC) scale, developed by Bhowmik et al. (30), was employed to assess health literacy among adolescents. The instrument consists of 10 items; each rated on a four-point Likert scale ranging from 1 (“not at all true”) to 4 (“absolutely true”). Scores are summed up to yield a total ranging from 10 to 40, with higher scores reflecting greater levels of health literacy. The scale has demonstrated acceptable reliability, with a Cronbach’s alpha of 0.759 reported by Bhowmik et al. (30), and in the present study, internal consistency was confirmed with a Cronbach’s alpha of 0.732.

#### Social network addiction scale

The Social Network Addiction Scale (SNAS), developed by Gökdaş and Kuzucu (31), was used to measure the level of social network addiction in adolescents and adults. The scale comprises 10 items distributed across three subscales: difficulty in control (4 items), negativity in social relations (3 items), and decrease in functioning (3 items). Each item is rated on a five-point Likert scale ranging from 1 (“never”) to 5 (“very often”), with higher total scores reflecting greater social network addiction and more pronounced negative effects on social and daily functioning. The scale’s reliability has been demonstrated using McDonald’s Omega, yielding an overall coefficient of 0.91, indicating excellent internal consistency. In the current study, the SNAS showed good reliability, with a Cronbach’s alpha of 0.790.

### Study procedures

#### Tool preparation and pilot study

The study instruments were translated into Arabic by bilingual experts fluent in both English and Arabic. The translation process for the original English scales followed the guidelines of Beaton et al. (32), including paraphrasing, forward and backward translation, and expert evaluation to ensure linguistic accuracy and cultural equivalence. Face validity was assessed through review by expert panels to verify that the translated instruments accurately represented the intended constructs within the Arabic cultural context. Feedback was also obtained from potential participants to evaluate the clarity, relevance, and cultural appropriateness of the questionnaire items. Internal consistency reliability for each scale was examined using Cronbach’s alpha.

Prior to data collection, a pilot study was conducted with 40 participants to assess the feasibility, validity, and reliability of the survey instruments and study procedures. The pilot findings revealed no concerns regarding the clarity or sequence of the questions, and participants reported that the survey was clear and easy to complete. Accordingly, no modifications were required, and the instruments were considered appropriate for use in the main study, supporting the overall rigor and validity of the research.

#### Data collection

Data were collected by trained researchers between February and April 2026 through the distribution of questionnaires to students during classroom sessions. To ensure anonymity, participants were asked not to write any personal identifying information on the questionnaires. Participation was voluntary, and students who chose not to participate were allowed to decline without any consequences. Completing the questionnaire required approximately 10–15 min. After completion, participants returned the questionnaires anonymously to sealed envelopes placed in a designated collection box to maintain privacy and confidentiality.

#### Ethical considerations

The Faculty of Nursing Research Ethics Committee at Beni-Suef University, Egypt, approved the study (Code: FNBSURECP/24022026178). Permission was also obtained from the directors of the selected institutions after explaining the study objectives. Since all participants were minors, written informed consent was obtained from their parents or legal guardians, and written assent was obtained from the children. Participants and their guardians received a full explanation of the study procedures, the voluntary nature of participation, and their right to withdraw at any time without penalty. The researchers protected participants’ privacy and confidentiality, and all collected data were handled confidentially.

### Statistical analysis

The data were analyzed using IBM SPSS Statistics version 27.0 (SPSS Inc., Chicago, IL, USA). Descriptive statistics (frequencies, percentages, means, and standard deviations) were used to characterize participant characteristics. When normality was evaluated using the standardized residuals’ histograms and normal probability (P–P) plots, the results showed a reasonably normal distribution. Independent-samples t-tests and ANOVA were used to examine group differences, while Pearson correlation was used to assess correlations between continuous variables. Multicollinearity was measured using data from the Variance Inflation Factor (VIF) and Tolerance. Because all tolerance values above the 0.10 criterion and all VIF values were less than the generally used cutoff of 5, multicollinearity was not deemed a threat (33, 34). The mediation study using Hayes’ PROCESS Macro (Model 4, version 4.2) with 5,000 bootstrap samples and 95% bias-corrected confidence intervals was conducted to examine whether health literacy mediated the relationship between health promotion (independent variable) and social network addiction symptoms (dependent variable). The mediation models were adjusted for the sociodemographic covariates that showed significant associations with social network addiction symptoms in the preliminary analyses, namely gender, mother’s educational level, and home internet access. Gender was coded as 0 = female and 1 = male. Mother’s educational level was entered as a categorical variable using dummy coding, with the lowest educational level serving as the reference category. Home internet access was coded as 0 = no and 1 = yes. Regression coefficients presented in the mediation analyses are unstandardized coefficients (*B*) and standardized coefficients (*β*), with their corresponding standard errors (SE), 95% confidence intervals (CI) (Table 1), and *p*-values. Statistical significance was established when the 95% bootstrap confidence interval for the indirect effect did not include zero. *p* < 0.05 was set as the cutoff point for statistical significance.

**Table 1:** Direct, indirect, and total effects of health promotion on social network addiction symptoms mediated by health literacy.

Models	*B*	*β*	*SE*	*t*	95% CI	*p*-value
MV Model						
Health promotion → health literacy	0.136	0.299	0.020	6.820	0.097 to 0.175	<0.001
Model summary *R*^2^ = 0.163, *F* = 22.867, *p* < 0.001						
DV Model						
Health promotion → social network addiction symptoms	−0.016	−0.020	0.037	−0.434	−0.088 to 0.056	0.664
Health literacy → social network addiction symptoms	−0.488	−0.277	0.081	−6.023	−0.647 to −0.328	<0.001
Gender (covariate)	−0.920	−0.051	0.821	−1.120	−2.534 to 0.694	0.263
Mother’s educational level (covariate)	−2.236	−0.227	0.445	−5.020	−3.111 to −1.360	<0.001
Internet access at home (covariate)	−7.129	−0.305	1.039	−6.863	−9.170 to −5.088	<0.001
Model summary *R*^2^ = 0.167, *F* = 18.761, *p* < 0.001						
Total effect						
Health promotion → social network addiction symptoms	−0.082	−0.103	0.036	−2.267	−0.154 to −0.011	0.024
Model summary *R*^2^ = 0.102, *F* = 13.378, *p* < 0.001						

Indirect effect	*β*	BootSE	BootLLCI	BootULCI
Health promotion → health literacy → social network addiction symptoms	−0.083	0.019	−0.122	−0.048

*B*, unstandardized coefficient; *β*, standardized coefficient; SE, standard error; *t*, *t*-value; CI, confidence interval; LLCI, lower limit of confidence interval; ULCI, upper limit of confidence interval; *p*, probability value; R^2^, coefficient of determination; *F*, *F*-statistic; MV, mediator variable model; DV, dependent variable model; BootSE, bootstrap standard error.

## Results

Table 2 presents the relationship between participants’ socio-demographic characteristics and social network addiction symptoms. The mean age of participants was 16.69 ± 0.85 years. No statistically significant difference was observed in social network addiction symptoms across age groups (*p* = 0.057), although participants aged 17–18 years reported slightly higher scores than those aged 15–16 years.

**Table 2:** Relationship between participants’ socio-demographic characteristics and social network addiction symptoms (*N* = 475).

Variables	Categories	*n*	%	Social network addiction symptoms	
				Mean ± SD	t/f (*p*-value)
Age	16.69 ± 0.85				
	15–16	199	41.9	29.17 ± 8.22	−1.888 (0.057)
	17–18	276	58.1	30.67 ± 8.71	
Gender					
	Male	155	32.6	31.21 ± 8.59	2.084 (0.038)
	Female	320	67.4	29.47 ± 8.46	
Residence					
	Rural	288	60.6	29.92 ± 8.90	−0.371 (0.711)
	Urban	187	39.4	30.22 ± 7.95	
Father’s working status					
	Yes	446	93.9	29.93 ± 8.63	−1.116 (0.268)
	No	29	6.1	31.75 ± 6.74	
Mother’s working status					
	Yes	179	37.7	30.08 ± 9.07	0.090 (0.929)
	No	296	62.3	30.01 ± 8.20	
Family type					
	Nuclear	304	64.0	30.42 ± 8.33	1.306 (0.192)
	Extended	171	36.0	29.36 ± 8.87	
Father’s educational level					
	No formal education	20	4.2	25.85 ± 5.09	2.120 (0.097)
	Primary	65	13.7	31.03 ± 8.68	
	Secondary	169	35.6	29.71 ± 8.76	
	University or above	221	46.5	30.38 ± 8.49	
Mother’s educational level					
	No formal education	39	8.2	31.07 ± 6.93	9.351 (<0.001)
	Primary	37	7.8	29.16 ± 8.90	
	Secondary	229	48.2	31.89 ± 9.02	
	University or above	170	35.8	27.51 ± 7.43	
Device type					
	Smartphone	433	91.2	30.30 ± 8.50	2.328 (0.099)
	Tablet	17	3.6	26.64 ± 8.93	
	Computer	25	5.3	27.92 ± 8.27	
Internet access at home					
	Yes	400	84.2	30.83 ± 8.52	4.737 (<0.001)
	No	75	15.8	25.85 ± 7.34	

*t*, Independent *t*-test; *f*, ANOVA test.

A significant difference was found by gender (*p* = 0.038), with males reporting higher levels of social network addiction symptoms compared to females. In contrast, residence, father’s working status, mother’s working status, and family type were not significantly associated with social network addiction symptoms (*p* > 0.05).

Regarding parental education, father’s educational level was not significantly related to social network addiction (*p* = 0.097). However, mother’s educational level showed a significant association (*p* < 0.001), with participants whose mothers had secondary education reporting the highest addiction scores, while those with university-educated mothers reported the lowest.

Device type was not significantly associated with social network addiction symptoms (*p* = 0.099), although smartphone users showed relatively higher scores. Notably, internet access at home was significantly associated with social network addiction symptoms (*p* < 0.001), with participants having home internet access reporting higher addiction levels compared to those without access.

Table 3 presents descriptive statistics and correlations among health promotion, health literacy, and social network addiction symptoms. The mean scores were 70.61 ± 10.69 for health promotion, 31.02 ± 4.85 for health literacy, and 30.04 ± 8.53 for social network addiction symptoms.

**Table 3:** Descriptive statistics and correlation between study variables (*N* = 475).

Variables	Mean ± SD	1	2	3
1. Health promotion	70.61 ± 10.69	1		
2. Health literacy	31.02 ± 4.85	0.327**		
		<0.001		
3. Social network addiction symptoms	30.04 ± 8.53	−0.117*	−0.229**	1
		0.011	<0.001	

*Correlation is significant at the 0.05 level (2-tailed). **Correlation is significant at the 0.01 level (2-tailed).

Pearson correlation analysis revealed a statistically significant positive correlation between health promotion and health literacy (*r* = 0.327, *p* < 0.001), indicating that higher levels of health literacy are associated with better health-promoting behaviors. In contrast, social network addiction symptoms were significantly and negatively correlated with both health promotion (*r* = −0.117, *p* = 0.011) and health literacy (*r* = −0.229, *p* < 0.001). These findings suggest that higher levels of social network addiction symptoms are associated with lower health literacy and poorer health promotion behaviors.

Table 1 and Figure 1 present the direct, indirect, and total effects of health promotion on social network addiction symptoms, with health literacy examined as a mediator. In the mediator model, health promotion had a significant positive effect on health literacy (*β* = 0.299, B = 0.136, SE = 0.020, *t* = 6.820, *p* < 0.001), explaining 16.3% of the variance.

**Figure 1:**
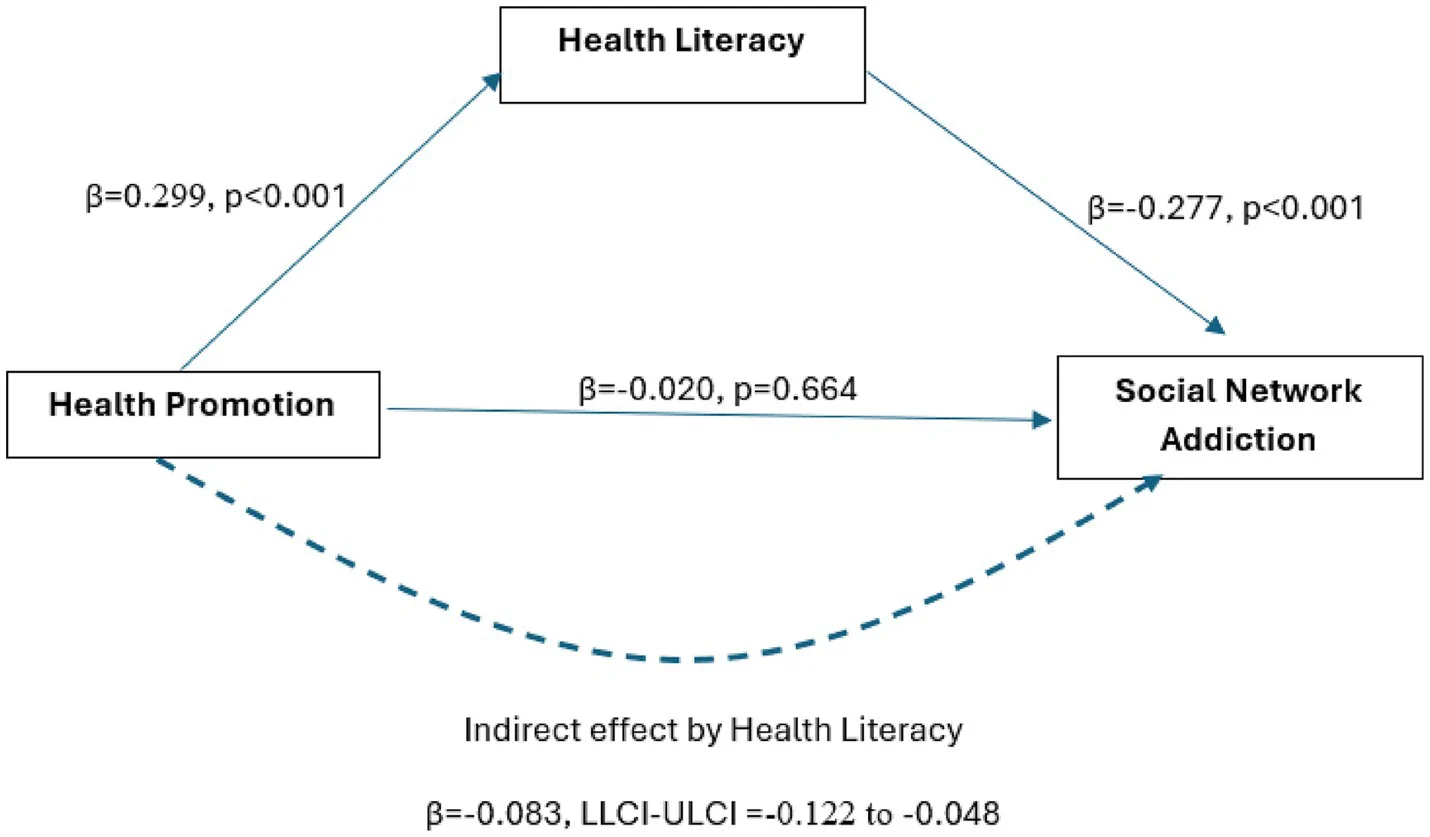
The mediating effect of health literacy on the relationship between health promotion and social network addiction symptoms.

In the direct effects model, health promotion was not a significant associated factor of social network addiction symptoms (*β* = −0.020, *p* = 0.664). However, health literacy showed a significant negative association with social network addiction symptoms (*β* = −0.277, *B* = −0.488, SE = 0.081, *t* = −6.023, *p* < 0.001), indicating that higher health literacy is associated with lower levels of social network addiction symptoms. Among the covariates, mother’s educational level (*β* = −0.227, *p* < 0.001) and internet access at home (*β* = −0.305, *p* < 0.001) were significant associated factors, while gender was not (*p* = 0.263). The model explained 16.7% of the variance in social network addiction symptoms.

The total effect of health promotion on social network addiction symptoms was statistically significant (*β* = −0.103, B = −0.082, SE = 0.036, t = −2.267, *p* = 0.024), explaining 10.2% of the variance. Bootstrapping analysis revealed a significant indirect effect of health promotion on social network addiction symptoms through health literacy [*β* = −0.083, BootSE = 0.019, 95% CI (−0.122, −0.048)], as the confidence interval did not include zero. These findings indicate that health literacy statistically explains the observed indirect association between health promotion and social network addiction symptoms. However, because of the cross-sectional design, causal mediation cannot be confirmed.

## Discussion

The present study explored the relationship between health promotion and health literacy and how health literacy may serve as an indirect pathway between health promotion and problematic social network use symptoms among adolescents. The results do not suggest a causal mechanism, but rather a cross-sectional relationship between participants who reported more health-promoting behaviors and higher health literacy, and between higher health literacy and lower social network addiction symptoms. This pattern is significant because it indicates that problematic social network use is not just digital behavior, but a part of a larger adolescent health profile that encompasses lifestyle practices, self-regulation, access to health information, and informed decision-making in digital environments.

The prevalence of social network addiction symptoms observed is in line with the growing concern of Social network addiction symptoms among adolescents in the world. The World Health Organization (WHO) has identified Social network addiction symptoms as a new adolescent health issue and has placed a strong focus on digital literacy as a tool to support young people to use social media safely (35). This is especially important in adolescence when participants are more sensitive to peer acceptance, more motivated to seek rewards, more prone to comparing themselves to others, and more likely to be exploring their identity. These developmental characteristics can make social networking sites particularly appealing, as they offer instant feedback, social proof, and ongoing interaction. Adolescents with poor offline coping abilities, poor daily routines, and poor digital self-management skills may therefore be more susceptible to unregulated or excessive social network use.

The negative correlation between health promotion and social network addiction symptoms can be understood in the context of the general function of healthy lifestyle behaviors in the self-regulation of adolescents. Health-promoting behaviors (physical activity, stress management, health responsibility, social support, life appreciation) could indicate a more structured and adaptive lifestyle pattern. Adolescents who engage in these behaviors may also have more structured time use, better emotional regulation, and stronger offline sources of support, which may decrease their need for social media to regulate their emotions, escape, or seek social reassurance. This interpretation aligns with the recent literature that suggests intrapersonal and interpersonal factors such as emotional regulation, self-control, peer relationships, and family support are linked to Social network addiction symptoms (36). These associations, however, should be interpreted with caution as the present study was cross-sectional. Another possibility is that teens who use social networks more negatively may not have the time, the motivation, or the psychological energy to participate in health-promoting activities.

The negative association between health literacy and social network addiction symptoms was stronger than the negative association between health promotion and social network addiction symptoms. The result indicates that in the digital realm, health literacy could be an especially important cognitive and behavioral asset. Adolescents who are more health literate may have an increased capacity to understand the health implications of excessive social media use, to critically assess information they encounter online, to identify unhealthy social media use, and to make more informed choices regarding screen time, sleep, privacy, and online interactions. Participants are exposed to health information, misinformation, commercial persuasion, social comparison, and algorithmically reinforced content in digital environments. In this context, health literacy is not just about understanding health information, but also about navigating the digital environment, evaluating information sources, safeguarding privacy, and interpreting information into healthier actions. This interpretation is supported by evidence that many children and adolescents have poor or problematic digital health literacy, especially regarding privacy protection, online navigation, and evaluating online information (37).

The statistical mediation model showed an indirect association, which gives additional insight into the possible association between these variables. The health promotion was positively related to health literacy, while health literacy was negatively related to social network addiction symptoms. This indicates that health-enhancing behaviors might be more strongly associated with reduced problematic social network use symptoms for adolescents with literacy skills that allow them to understand health risks and manage digital behaviors. In real terms, healthy behaviors may not be enough if adolescents are not knowledgeable, judgemental or have the self-regulatory skills to identify problematic use of the internet. The discovery also aligns with previous research that found that eHealth literacy, mental health literacy, and social media addiction were negatively associated in adolescents (38). However, the present finding should be interpreted as an indirect statistical association, rather than as evidence that health promotion leads to improvements in health literacy, or that health literacy causally decreases symptoms of social network addiction.

Although the final model demonstrated a significant indirect association between health promotion, health literacy, and social network addiction symptoms, it explained 16.7% of the variance in social network addiction symptoms. This indicates that other important factors not included in the current model may contribute to adolescents’ problematic social network use behaviors. Previous research has identified several potential determinants, including the amount of daily screen time, psychological distress (such as anxiety, depression, and stress), sleep quality, parental monitoring and supervision, peer influence, and the specific type of social media platforms used. These factors may interact with individual health-related characteristics and digital behaviors, influencing adolescents’ vulnerability to developing social network addiction symptoms. Future studies should incorporate broader psychosocial, behavioral, and technology-related variables to provide a more comprehensive understanding of the factors associated with social network addiction symptoms among adolescents.

The results have implications for school-based and community-based health promotion programs. There is a need to go beyond just limiting access and decreasing screen time to address problematic social network use. While time management is important, adolescents also need to learn skills that will help them understand the negative consequences of excessive time management and how online content can impact their emotions and behaviors, and how to develop healthier offline habits. The inclusion of digital health literacy, emotional regulation, sleep hygiene, physical activity, online privacy, critical appraisal of online information, and responsible social media use in health education programs could therefore be beneficial. The results of health education interventions indicate a possible effect on reducing the time spent on the internet and internet addiction among adolescents, but there is little evidence specifically targeting social media addiction (39). Likewise, a co-designed digital health literacy educational tool has been demonstrated to enhance adolescents’ skills and confidence in recognizing credible health information found online (40). The results indicate that there is a need for practical, age-appropriate, and participatory educational strategies, rather than only restrictive ones.

The gender and mother’s education and home internet association also need to be interpreted with caution. The higher scores on social network addiction symptoms for males may be related to the activities they engage in online, how they interact with peers, their gaming-related use of social networks, or their risk-taking behaviors. This correlation with the mother’s education level could be due to variations in supervision, communication, health advice, and online boundaries at home. Having access to the internet at home was also linked to higher symptom scores, as would be predicted, as more time spent using the internet would lead to more opportunities for prolonged or unsupervised use. But the access to the internet should not be taken to mean that it is harmful. The real question is whether adolescents are sufficiently guided, have digital boundaries, communicate with their families, and have literacy skills that allow them to navigate online platforms safely and effectively.

Overall, the present findings support an integrated approach to adolescent digital wellbeing. Health promotion programs should not treat problematic social network use as only a behavioral discipline issue or a technology-use issue. Instead, it should be addressed as part of adolescent health development, including lifestyle balance, self-regulation, social support, sleep protection, and health literacy. The National Academies of Sciences, Engineering, and Medicine emphasized the importance of digital media literacy, online safety, health-care provider engagement, and balanced policies to protect adolescent health in the social media era (41). In line with this perspective, school and community health nurses can play a central role by screening for problematic digital behaviors, educating adolescents and parents, promoting healthy routines, and supporting students in developing the literacy skills required to navigate online environments safely.

### Strengths and limitations

This study has several strengths. First, it addresses an important and timely public health issue in Egypt, where evidence on health promotion, health literacy, and problematic social network use symptoms among adolescents remains limited. Second, the study included an adequate sample size and used a multistage random sampling technique, which strengthened the representativeness of the school-based sample. Third, standardized instruments were used to assess the main study variables, and the statistical mediation model allowed exploration of a possible indirect association between health-promoting behaviors and social network addiction symptoms through health literacy.

Despite these strengths, several limitations should be considered. The cross-sectional design prevents conclusions about causality, temporal ordering, or causal mediation. Therefore, the findings should be interpreted as exploratory associations rather than evidence that health promotion or health literacy reduces problematic social network use symptoms. Longitudinal and intervention studies are needed to clarify the direction and causal nature of these relationships.

Another limitation is the reliance on self-reported questionnaires administered at a single time point. This may have introduced recall bias, social desirability bias, and common-method bias, which could inflate the observed associations among health promotion, health literacy, and social network addiction symptoms. In addition, the study did not collect objective digital-use data, such as device-recorded screen-time reports, app-use logs, or platform-specific usage records. As a result, the findings reflect adolescents’ self-reported symptoms rather than objectively verified patterns of social media use.

The study was also conducted in four schools within a single Egyptian governorate, which may limit the generalizability of the findings to adolescents in other geographical, cultural, or educational contexts. Moreover, possible school-level clustering was not accounted for in the statistical analyses. Students within the same school may share similar educational, social, and digital environments, which could influence standard errors and significance testing. Future studies should consider multilevel modeling or cluster-robust analytic approaches when students are nested within schools.

Finally, although the model adjusted for gender, mother’s educational level, and home internet access, several potentially important confounding variables were not measured or included in the analysis. The final model explained 16.7% of the variance in social network addiction symptoms, suggesting that additional psychological, behavioral, social, and technology-related factors may contribute to adolescents’ problematic social network use symptoms. These unmeasured factors may include daily screen time, sleep quality, psychological distress, parental monitoring, educational stress, peer influence, and platform-specific patterns of social media use. Such factors may be associated with health-promoting behaviors, health literacy, and problematic social network use symptoms, and their omission may have influenced the observed associations. Future longitudinal studies should incorporate these variables to improve the explanatory power of the model and provide a more comprehensive understanding of the complex factors influencing adolescent digital health behaviors.

### Nursing implications

The results have relevance for school and community health nurses. Using social media is an aspect of social life that nurses should evaluate as part of their regular health promotion and school health programs. In addition to the amount of screen time, screening should also include loss of control, functional impairment, sleep issues, adverse impact on schoolwork, and emotional dependency on social media. School nurses can develop educational initiatives to address digital health literacy, healthy technology use, stress reduction and management, sleep hygiene, physical activity, online privacy, and assessing online health information, as well as health promotion.

Prominent digital health literacy in the curriculum of pediatric nursing, school health nursing, and community health nursing should also be incorporated into the nursing education training of nursing educators. This would help equip future nurses with the skills to provide future care to adolescents facing technology-related behavioral risks. Nurses should also offer parenting education on topics such as supervision, communication, digital boundaries with the Internet, and healthy digital behaviors at home.

## Conclusion

The present cross-sectional study found that health promotion, health literacy, and social network addiction symptoms were significantly interrelated among adolescents. Health promotion was positively associated with health literacy, and both health promotion and health literacy were negatively associated with social network addiction symptoms. In the exploratory statistical mediation model, health literacy accounted for a significant indirect association between health promotion and social network addiction symptoms, while the direct association between health promotion and social network addiction symptoms was not statistically significant after including health literacy.

## Data Availability

The raw data supporting the conclusions of this article will be made available by the authors, without undue reservation.
